# Biogeographical variation in antimicrobial resistance in rivers is influenced by agriculture and is spread through bacteriophages

**DOI:** 10.1111/1462-2920.16122

**Published:** 2022-07-07

**Authors:** Tilde Andersson, Aiko D. Adell, Andrea I. Moreno‐Switt, Peter Spégel, Charlotta Turner, Søren Overballe‐Petersen, Kurt Fuursted, Rolf Lood

**Affiliations:** ^1^ Department of Clinical Sciences Lund University Lund Sweden; ^2^ Escuela de Medicina Veterinaria, Facultad de Ciencias de la Vida Universidad Andres Bello Santiago Chile; ^3^ Millennium Initiative for Collaborative Research On Bacterial Resistance (MICROB‐R) Santiago Chile; ^4^ Escuela de Medicina Veterinaria, Facultad de Agronomía e Ingeniería Forestal, Facultad de Ciencias Biológicas y Facultad de Medicina Pontificia Universidad Católica de Chile Santiago Chile; ^5^ Department of Chemistry Lund University Lund Sweden; ^6^ Statens Serum Institute Bacterial Reference Center Copenhagen Denmark

## Abstract

Antibiotic resistance is currently an extensive medical challenge worldwide, with global numbers increasing steadily. Recent data have highlighted wastewater treatment plants as a reservoir of resistance genes. The impact of these findings for human health can best be summarized using a One Health concept. However, the molecular mechanisms impacting resistance spread have not been carefully evaluated. Bacterial viruses, that is bacteriophages, have recently been shown to be important mediators of bacterial resistance genes in environmental milieus and are transferrable to human pathogens. Herein, we investigated the biogeographical impact on resistance spread through river‐borne bacteriophages using amplicon deep sequencing of the microbiota, absolute quantification of resistance genes using ddPCR, and phage induction capacity within wastewater. Microbial biodiversity of the rivers is significantly affected by river site, surrounding milieu and time of sampling. Furthermore, areas of land associated with agriculture had a significantly higher ability to induce bacteriophages carrying antibiotic resistance genes, indicating their impact on resistance spread. It is imperative that we continue to analyse global antibiotic resistance problem from a One Health perspective to gain novel insights into mechanisms of resistance spread.

## INTRODUCTION

Resistance to antibiotics in clinically relevant bacteria has been documented for several decades (Davies, 1996). Introduction of early antibiotics developed in the 1940s, including penicillin (β‐lactam), led to resistance almost instantaneously (Kong et al., 2010). Resistance genes for antibiotics are present in environmental microbial populations even before clinical usage of antibiotics is initiated (Cantón, 2009). However, spread of such resistance among human pathogens is not instigated until a selective pressure (e.g. usage of antibiotics) is added—triggering development and selection for clinically resistant bacteria (Davies, 1996). Antibiotic resistance can be spread through horizontal gene transfer; through conjugation, transduction and transformation. Conjugation (e.g. cell‐to‐cell transfer) has historically been demonstrated to be of high importance due to the high success rate of transfer due to the close contact between the donor and recipient (Virolle et al., 2020). In addition, transduction, where bacteriophages transfer DNA from one donor bacteria to another recipient bacteria, can be highly efficient and play an important role in spread of antibiotic resistance (Muniesa et al., 2011, 2013), though conjugation is likely still the dominant force in spread of resistance (Che et al., 2021). In particular, transduction has been demonstrated in polluted water and wastewater treatment plants (WWTPs) (Bouki et al., 2013; Rizzo et al., 2013). These waters have vast amounts of bacteria and other microbes transiting them on a daily basis, including resistance genes in various forms (resistant bacteria, free DNA, phage particles carrying resistance genes) (Parsley et al., 2010) enabling a high probability of gene transfer within this bacterial community. Bacteriophages carrying resistance genes have, however, not only been identified within WWTPs, but are common in the environment, and are suggested to function as a reservoir of both known and unknown antibiotic resistance genes (Balcazar, 2014; Moon et al., 2020). Directed screens have identified resistance genes particularly towards tetracyclines and β‐lactamases often being carried by environmentally isolated phages (Anand et al., 2016; Lekunberri et al., 2017; Sala‐Comorera et al., 2021), with resistance genes prevalence within the phage population sometimes outnumbering the prevalence in bacteria (Zhang et al., 2019). Similarly, bacteriophages have been indicated as a putative mediator of antibiotic resistance in agro‐food systems (Jebri et al., 2021; Yang et al., 2020). Several recent studies have employed metagenomics approaches rather than directed amplicon screens. These methods allow for taxonomic identification of sample, but also add information in terms of functional genes. Furthermore, metagenomics analyses have a higher depth compared to 16S rRNA sequencing, meaning that less abundant microbial taxa can be detected (Durazzi et al., 2021). Very limited data are available on absolute resistance gene quantities within these populations and environments, including an absence of mechanistic insights into what confers such presence of resistance genes.

Furthermore, the prevalence of subclinical levels of antibiotics and other stressors present in low concentrations in polluted water further increases the selection of antimicrobial‐resistant bacteria in the environment (Li et al., 2009). The additional stresses that bacteria are exposed to in polluted water may also lead to induction of prophages (Motlagh et al., 2015), further increasing the probability of gene transfer. In vivo murine experiments have confirmed phage activation when exposed to subclinical levels of antibiotics, leading to not only increased phage induction but also to unique resistome patterns carried by these phages based on the stressor used (Modi et al., 2013). Thus, particular classes of antibiotics differentially impact bacteria‐phage networks and phage transduction. It is well established that the majority of mobile resistance genes found in clinically important pathogens have originated in environmental bacteria and have subsequently undergone horizontal gene transfer (von Wintersdorff et al., 2016). Although it is not historically possible to know exactly where this has taken place, the co‐residence of both pathogens and the environmental carriers of resistance genes can be found in polluted water (Lood et al., 2017; Rizzo et al., 2013).

Several industrialized countries have access to systems reducing spread of resistant bacteria in polluted water, with less focus placed on reducing spread of resistance gene. Human health care is not solely responsible for development and spread of antibiotic resistance, an idea encompassed under the One Health umbrella. This concept describes the role of other industries (e.g. veterinary, agriculture, aquaculture, livestock, etc.) and how they may significantly impact the development of clinically relevant pathogens (Destoumieux‐Garzón et al., 2018; Mackenzie & Jeggo, 2019). In particular, in countries with high levels of agriculture and aquaculture these influences may be detrimental (Cabrera‐Pardo et al., 2019). Veterinary usage of antibiotics widely exceeds that of human medicine, with more than 95% of several important antibiotics used within industries other than for human health (Millanao et al., 2018). The trend is similar over several sectors, with for example the usage of antimicrobials in aquaculture more than doubling in Chile, from 143 tons in 2010 to 380 tons in 2016 (Miranda et al., 2018). This over‐usage of antibiotics has been shown to impact development of resistance both in environmental and human‐specific pathogens (Millanao et al., 2018), with increased prevalence of resistance genes prevalence more than 100 m downstream of aquaculture farms (Bueno et al., 2019). Water polluted with antibiotics (or other stressors) would thus serve as an inducer of bacteriophages, facilitating horizontal gene transfer of resistance genes. Recent studies have also highlighted that the mere evolutionary pressure exerted by phages on bacteria can drive exchange of genetic material between bacteria (LeGault et al., 2021).

While increasing numbers of studies have highlighted the need for a reduced usage of antibiotics, there is a lack of knowledge regarding the prevalence of antimicrobial resistance genes in rivers in Latin America and their impact on the environment and health (Leon‐Felix et al., 2020). Several studies have highlighted the prevalence of resistance genes within both bacteria and bacteriophages isolated from river samples, as well as how this varies due to biogeography, and how phages can transduce this resistance. Herein, we confirm these findings and add additional mechanistic insight in how this resistance spread is modulated by environmental stressors in a One Health setting.

## EXPERIMENTAL PROCEDURES

### Study area

**FIGURE 1:**
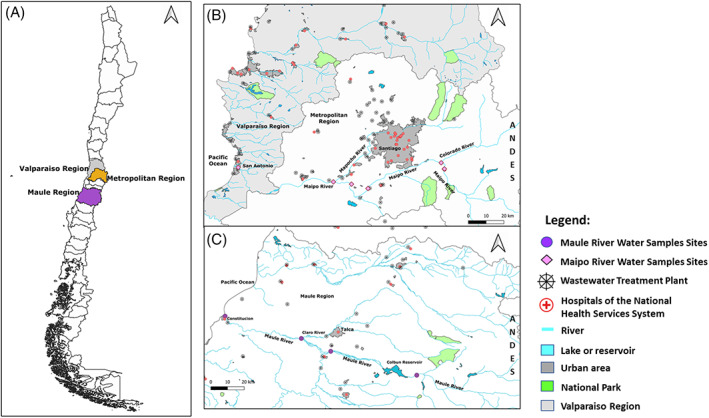
Geographical overview of isolated samples. Samples were isolated from river Maipo and Maule, crossing Chile in the Metropolitan, Valparaiso, and Maule region (A). Details regarding river Maipo and Maule are given in (B) and (C), respectively

Water samples were collected along the course of Maipo and Mapocho rivers, both located in the central macrozone of Chile. Maipo river crosses the metropolitan and V regions, while the Maule river crosses the VII region of the country. Maipo river is characterized by being the primary source of drinking water for the metropolitan region, supplying approximately 70% of the current demand for drinking water and 90% of the irrigation demands (Ministerio del Medio Ambiente, 2004). Maule river provides drinking water to 1667.07 hectares and has an irrigation surface of 118,263 hectares. The Maipo and Maule rivers share similar zones of land use, allowing a comparison between land use zones along both river courses (Figure 1).

The land use for each river was categorized based on the information provided by the Land Use Atlas of the Chilean Military Geographic Institute as (i) Environmental, (ii) Agriculture, (iii) Urban Zone and (iv) Livestock in Maipo river and livestock and forestry in Maule river. Sampling sites were selected to represent a land use described above. All sampling points were georeferenced using Google Map.

### Collection of material

Water was collected from the two Chilean rivers Maipo (eight sites; two sites being pre/post‐WWTPs) and Maule (four sites) during four different time periods (April 2018, August 2018, January 2019, April 2019). A total of 20 L river water was collected at each site per sampling effort using sterile plastic containers. Each container was rinsed three times in the river water before collecting the sample. The sample was collected close to the river shore, submerging the container at least 10 cm from the surface. River water samples were also collected before and after the discharge of treated wastewater from the two WWTPs located in the course of the Maipo River. Immediately after the samples were collected, the containers were placed in a cooler with icepacks and/or ice and transported to the Universidad Andres Bello laboratory on the same day to minimize losses, pollution and degradation of the samples. Samples were moved to a 4°C fridge within 24 h where they were aliquoted and then kept at −20°C. Details regarding the sampling sites can be found in Table 1 and Figure 1.

**TABLE 1:** Water sample information

River site	River	Distance from river source (km)	Region	Coordinates
MAU1	Maule	37	Environment	−35.714739; −71.122784
MAU2	Maule	94	Agriculture	−35.555387; −71.695976
MAU3	Maule	117	City	−35.467973; 71.894112
MAU4	Maule	182	Agriculture	−35.320605; −72.399858
MAI1	Maipo	56^a^	Environment	−33.634361; −70.355062
MAI2	Maipo	56^b^	City	−33.633846; −70.354109
MAI3	Maipo	67	Environment	−33.596716; −70.373771
MAI4	Maipo	122^a^	Agriculture	−33.752706; −70.818370
MAI5	Maipo	122^b^	City	−33.753288; −70.818832
MAI6	Maipo	136	Agriculture	−33.726965; −70.919111
MAI7	Maipo	143	City	−33.713014; −71.029048
MAI8	Maipo	216	Agriculture	−33.624822; −71.607586

^a^ Pre‐WWTP.

^b^ Post‐WWTP.

### Sample processing for DNA extraction

To detect bacteriophage and bacterial DNA in Maipo and Maule river water, 20 L of the water samples were concentrated to 200 ml using hollow‐fibre ultrafiltration system as described elsewhere (Bambic et al., 2011; Hill et al., 2005), using B Braun DIACAP HIPS20 haemodialysis filters. The final sample was concentrated ca. 100×. Samples were processed to purify bacterial and bacteriophage DNA. For bacterial DNA, ultrafiltrated samples were centrifugated at 10,000*g* for 10 min and the pellet was used to purify DNA using the PureLink Microbiome DNA Purification kit (Invitrogen, Waltham, MA, USA) following the manufacturer's instructions. For bacteriophage DNA, samples were lightly sonicated for 30 s to release bound bacteriophages and were centrifugated at 25,000*g* for 20 min and filtered using a 0.22 μm syringe‐attached filter. A Phage DNA isolation kit (Norgen Biotek, Thorold, ON, Canada) was used for DNA purification, according to the manufacturer's instructions, including addition of DNAse I and heat‐inactivation thereof. DNA was quantified with MaestroNano spectrophotometer (Maestro, Korea) and with Qubit fluorimeter (Life Technologies, Carlsbad, CA, USA).

### 16S/18S amplicon‐based microbiome analysis

Amplicon‐based microbiome analysis was done as previously described (Ring et al., 2017), and as illustrated in Figure S1. DNA was amplified using a two‐step PCR using custom 341F/806R primers targeting the V3–V4 16S regions, and three primer sets targeting the hyper‐variable regions V3–V4 of the 18S rDNA gene. Library preparation was performed by Nextera XT DNA Library Preparation (Illumina Inc., San Diego, CA, USA), and Illumina sequencing was performed on the MiSeq system (Illumina) according to the manufacturer's instructions using the V2 Reagent Kit.

### Oxford nanopore sequencing for resistance genes

DNA was prepared for sequencing using Oxford Nanopore Technologies' Rapid PCR Barcoding Kit (SQK‐RPB004) with the following modifications to the manufacturer's instructions: Double volume of template DNA and ‘FRM’, as well as 25 PCR cycles instead of 14. Pooled DNA libraries were sequenced in R9.4.1 flow cells (FLO‐MIN106) in a MinION (Oxford Nanopore Technology, Oxford, UK) connected to a MinIT with MinIT Release 19.12.5 (MinKNOW Core 3.6.5, Bream 4.3.16, Guppy 3.2.10). Samples were pooled, using 8–16 barcode samples per MinION run. Raw fast5‐reads were basecalled to fastq‐files with the ‘Fast’ configuration of the algorithm. Basecalled reads were analysed with the mapping tool KMA (K‐Mer Aligner) (Clausen et al., 2018) version KMA‐1.2.22 with the following parameters adapted for nanopore data: ‘‐mem_mode ‐mp 20 ‐mrs 0.0 ‐bcNano ‐and’. KMA were used for mapping against the following databases https://www.arb‐silva.de/no_cache/download/archive/release_132/Exports/Archaea (16S & 18S rRNA from bacteria, archaea and eukarya), https://www.cbs.dtu.dk/public/CGE/databases/KmerFinder/version/20190108_stable/ (Fungi, plasmids and protozoa), https://www.cbs.dtu.dk/public/CGE/databases/KVIT/version/20190513/ (viruses), as well as ResFinder database (2020‐04‐08) (Bortolaia et al., 2020) and PlasmidFinder (2020‐04‐02) (Carattoli & Hasman, 2020).

### Bioinformatics analysis of 16S/18S amplicon‐based microbiome sequence data

Bioinformatic analysis was done using BION (http://box.com/bion), a newly developed analytical semi‐commercial open‐source package for 16S and 18S rRNA and other reference gene analysis, classifying mostly to species level. The pipeline accepts raw fastq illumine sequences and includes steps for de‐multiplexing, primer‐extraction, sampling, sequence‐ and quality‐based trimming and filtering, de‐replication, clustering, chimaera‐checking, reference data similarities and taxonomic mapping and formatting. Non‐overlapping paired reads are allowed for analysis.

### Statistics of sequence data

Analysis of microbiome composition was performed in R version 4.0.32020‐10‐10 using the packages phyloseq v.1.24.2 and vegan v.2.5‐2. A 16S rarefaction threshold was set at 2000 reads for prokaryotics and an 18S rarefaction cut‐off at 100 reads for eukaryotics. Figures were created using ggplot2 v.3.2.0 and plotly v.4.8.0. In the bar plot, taxa were merged to genus level by agglomerating counts within each genus. Within‐sample (alpha) diversity, as well as relative abundances of individual genera, were compared between groups with Mann–Whitney rank‐sum tests and adjusted for multiple testing using Bonferroni correction. Differences between groups (beta‐diversity) were assessed with bar plots and principal coordinates analysis plots using Bray–Curtis distances and tested with a permutational multivariate analysis of variance, ‘*adonis’* from the package ‘*vegan’* v.2.5‐2.

### ddPCR amplification of resistance genes

A PCR reaction was prepared according to BioRad's instructions for 2×ddPCR Supermix for probes (no dUTP) instructions (BioRad). Droplets were prepared and analysed in a QX200 ddPCR system and evaluated with quantasoft 1.7. The PCR reaction was conducted in a BioRad C1000 thermal cycler, following standard cycling settings. Primers used for the reactions can be found in Table 2. For phage DNA, presence of 16S rRNA was routinely investigated to verify that only low levels could be detected.

**TABLE 2:** ddPCR primers used in the study

Target	Forward primer	Reverse primer	Probe
16S	AGAGTTTGATCCTGGCTCAGGA	CGTGTTACTCACCCGTCCG	CGCTGGCGGCGTGCCTAATACATGC
CTXM1	ACAGTACAGCGATAACGTGG	GAATGGCGGTGTTTAACGTC	GCGGCCCGGCTAGCGTCACC
CTXM9	GACTGTGGGTGATAAGACCG	TGTTGCGGCTGGGTAAAATA	GCAGGGTCGTGCGCCGCTGG
TetA	TTGAACGGCCTCAATTTCCT	GATGAAGAAGACCGCCATCA	GCATGACCGTCGTCGCCGCCC
TetM	TGCAAGAAAAGTATCATGTGGAG	AAACCGAGCTCTCATACTGC	TGCCGCCAAATCCTTTCTGGGCTTCCA

### Phage induction assay

*Escherichia coli* expressing the *recA*‐GFP reporter system (Horizon Discovery, PEC3876‐202384786) was cultured in LB supplemented with 25 μg/ml kanamycin and incubated o/n at 37°C, 200 rpm. A fresh culture diluted 1:9 in LB was prepared, and water samples were added to a final concentration of 50%. Samples were incubated at 37°C, 50 rpm, for 12 h before being analysed on a Victor fluorescent reader (485/530 nm). All samples were analysed in biological quadruplicates.

### Statistical tests

Differences between groups were assessed by ANOVA, with Tukey's test *post hoc* using the software SPSS. Levels of bacterial and phage resistance genes *tetA* and *tetM* were stratified as present (+) or absent (−) using the detectable level of the assay as a cut‐off. All data were analysed using the non‐parametric Mann–Whitney *U* test with *p* < 0.05 considered statistically significant, except comparing the prokaryotic and eukaryotic abundance between samples where *p* < 0.01 was considered significant.

## RESULTS

### Sampling site and strategy allow for a biogeographical resolution of the results

The rivers Maipo and Maule represent two of the main rivers in central Chile, crossing the country from the mountains in the east to the ocean in the west, thus traversing the country in its entirety and passing different geographical regions (e.g. urban, suburban, agriculture) as well as before and after WWTPs. With samples from different seasons, a temporal dimension of the samples could be added for the analysis. For details regarding sampling localization, see Table 1 and Figure 1.

### The examined rivers display significant difference in prokaryotic and eukaryotic diversity

**FIGURE 2:**
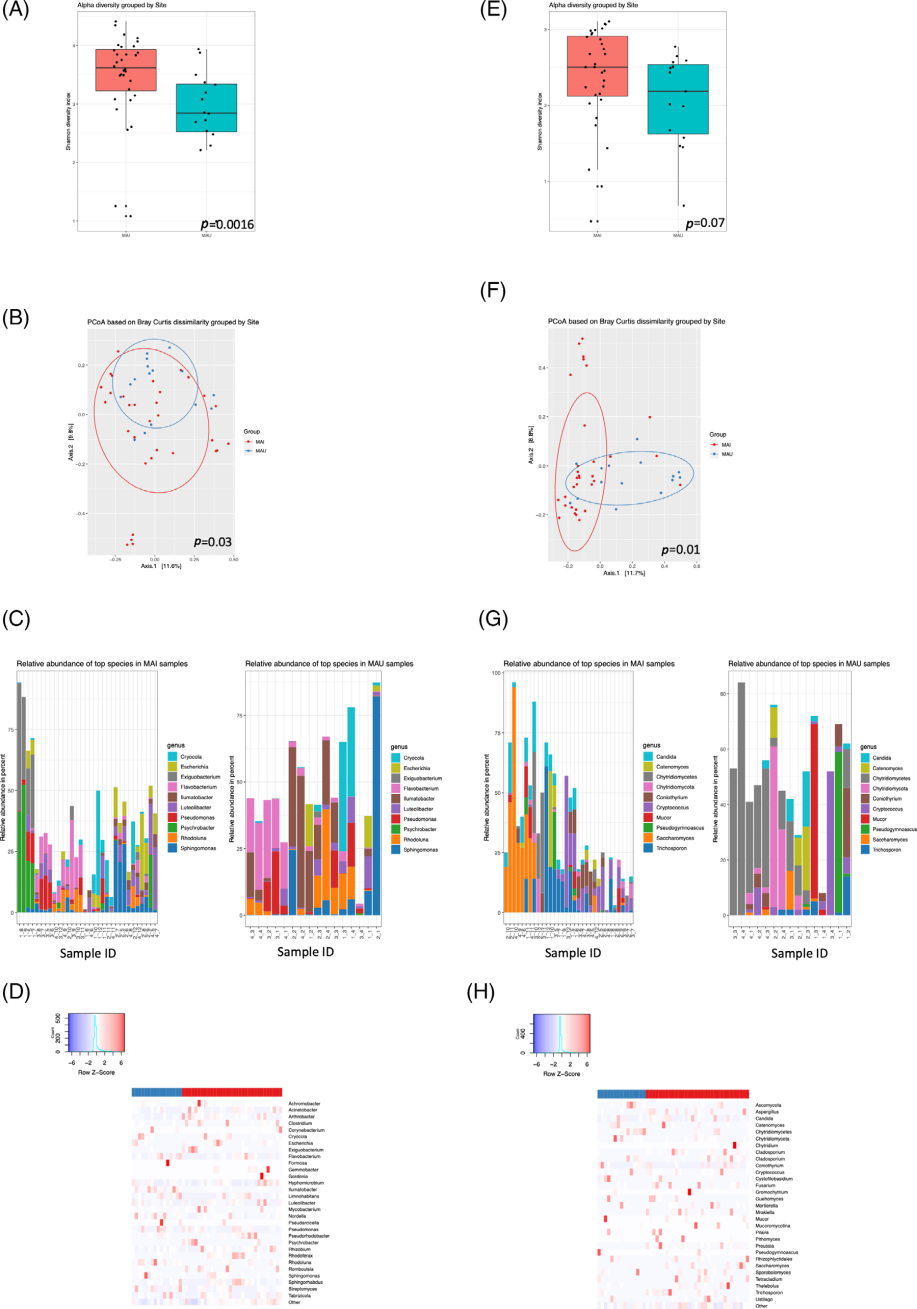
Microbial diversity varies between rivers. Water collected from the two Chilean rivers Maipo (MAI) and Maule (MAU) was analysed in terms of prokaryotic alpha (Shannon's Diversity Index) (A) and beta (Bray–Curtis dissimilarity analysis) (B) diversity based on Illumina 16S/18S sequencing. The top 10 most common prokaryotic genera identified in 48 samples from river Maipo and river Maule as detected by heat map analysis (C) as well as presented as abundance of the prokaryotic flora (D). Eukaryotic alpha (Shannon's Diversity Index) (E) and beta (Bray–Curtis dissimilarity analysis) (F) diversity was similarly plotted, with prevalence in river Maipo and Maule (G) with a corresponding heat map (H). Sample IDs are labelled according to *x*–*y*, where *x* is time point (e.g. 1–4) and *y* is location (e.g. MAI1‐8, MAU1‐4)

River Maipo displayed a significant higher within‐sample (alpha diversity) and between‐sample (beta diversity) in prokaryotic content compared to river Maule [Figure 2(A,B)]. Specifically, *Curvibacter* was significantly more abundant in river Maule compared to river Maipo (*p* = 0.0002) while the genus *Sulfurovum*, *Mezorhizobioum*, *Thermomonas*, *Ornithinibacter*, *Thiobacillus*, *Arthrobacter*, *Paracoccus* and *Devosia* were significantly more abundant in river Maipo compared to river Maule (*p* = 0.0003–0.001) [Figure 2(C,D)]. Eukaryotics showed less diversity between the two rivers, with only significance in beta diversity [Figure 2(E,F)], with *Chytridiomycetes* significantly more abundant in river Maule (*p* = 0.001) and *Preussia* more abundant in river Maipo (*p* = 0.005) [Figure 2(G,H)].

### River sites as a determinant for microbial composition

**FIGURE 3:**
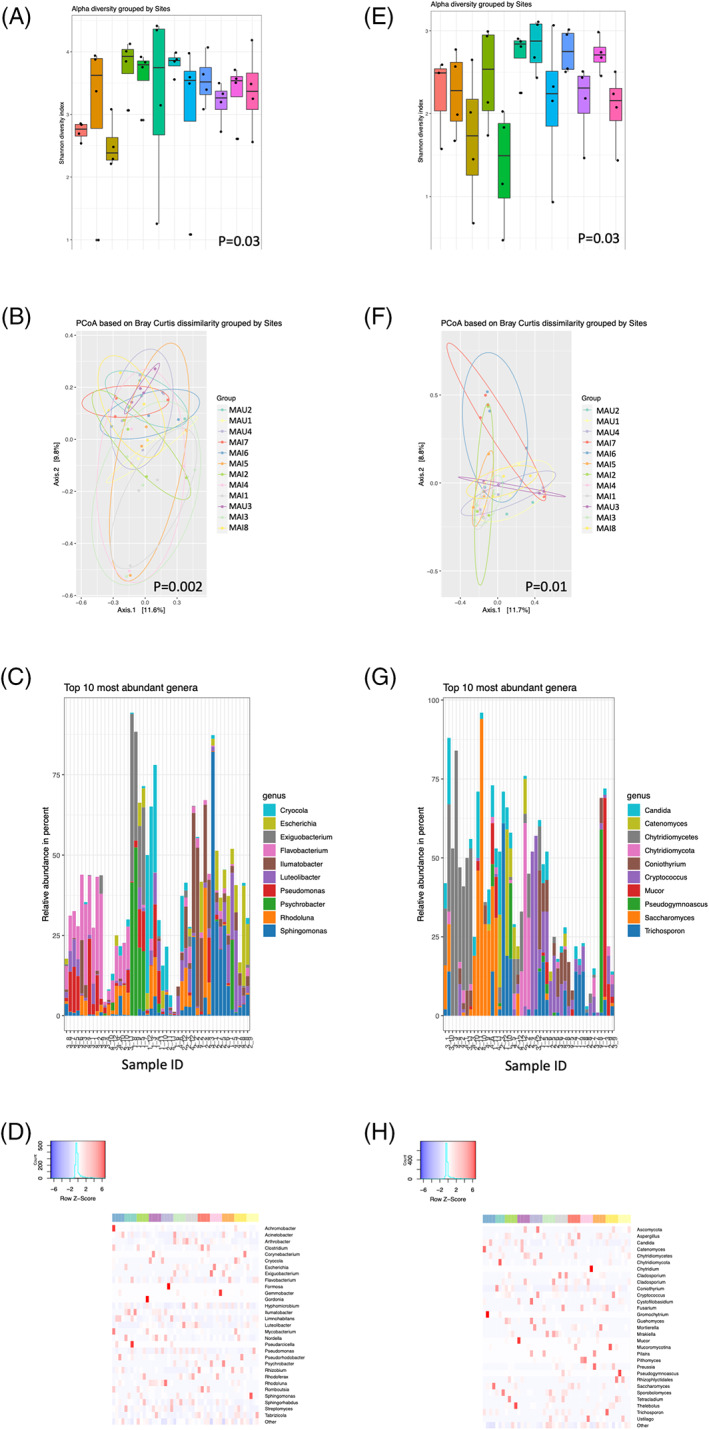
River site is a determinant for microbial diversity. Different river sites from both rivers (Maipo, *n* = 8; Maule, *n* = 4) were characterized for prokaryotic alpha (Shannon's Diversity Index) (A) and beta (Bray–Curtis dissimilarity analysis) (B) diversity. The top 10 most common prokaryotic genera as detected by heat map analysis (C) as well as presented as abundance of the prokaryotic flora (D). Similarly, eukaryotic alpha (Shannon's Diversity Index) (E) and beta (Bray–Curtis dissimilarity analysis) (F) diversity as well as prevalence in the river sites (G) were plotted with a corresponding heat map (H). Sample IDs are labelled according to *x*–*y*, where *x* is time point (e.g. 1–4) and *y* is location (e.g. MAI1‐8, MAU1‐4)

In general, the alpha diversity was highly comparable between the 12 river sites, with no significant difference in the content [Figure 3(A,E)]. Beta diversity was significantly different, both regarding prokaryotics [*p* = 0.002; Figure 3(B)] and eukaryotics [*p* = 0.01; Figure 3(F)]. The abundance of prokaryotics and eukaryotics between the different river sites was comparable with no significant differences [Figure 3(C–H), Supplementary Figures 2–3]. Using metagenomics nanopore sequencing, several viruses could also be identified (at query ID >80% and Template coverage >20%) in the river sites, mostly including human endogenous retrovirus, and ‘Sewage‐associated circular DNA virus’ (data not shown).

### Microbial biodiversity in rivers change over time

**FIGURE 4:**
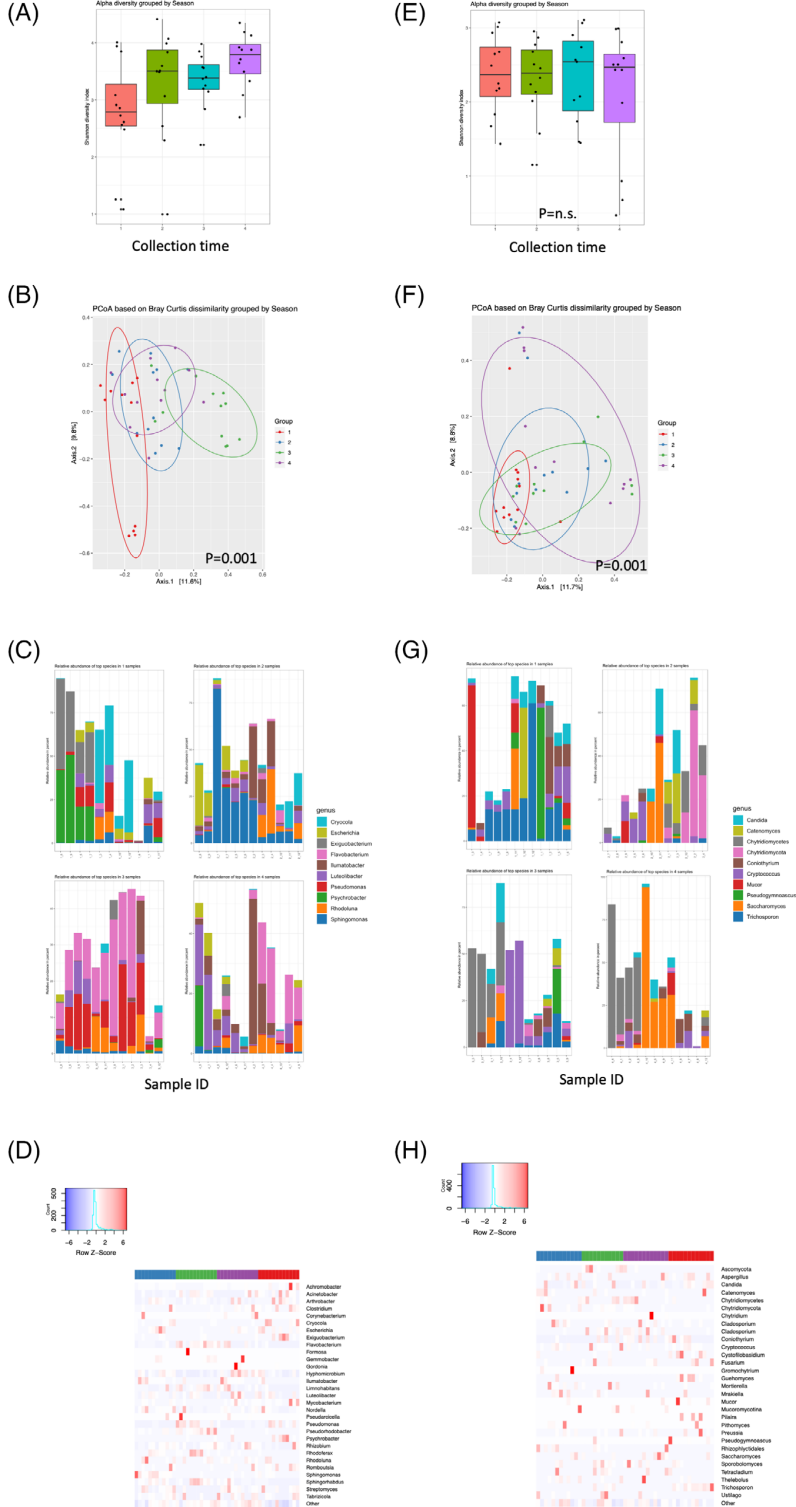
Temporal changes affect microbial biodiversity in rivers. River samples were collected during different time points, and prokaryotic and eukaryotic diversity changes monitored through changes in the 16S and 18S rRNA composition. Alpha diversity (Shannon's Diversity Index) for prokaryotes (A) and eukaryotes (E), beta diversity (Bray–Curtis dissimilarity analysis) for prokaryotes (B) and eukaryotes (F), the top 10 most commonly identified prokaryotes (C) and eukaryotes (G) during the different collection time points based on heat map analysis were plotted and summarized in a heat map representing the abundance for prokaryotes (D) and eukaryotes (H). Note that the prevalence plot and heat map is identical to Figure 2(C,D) and Figure 2(G,H), but the analysis differs. Sample IDs are labelled according to *x*–*y*, where *x* is time point (e.g. 1–4) and *y* is location (e.g. MAI1‐8, MAU1‐4)

Temporal changes were associated with significant changes in alpha diversity for prokaryotes between time points 18.04 and 19.04 [*p* = 0.01, Figure 4(A)], with only limited changes in eukaryotic alpha diversity [Figure 4(E)]. Beta diversity was significantly different (*p* = 0.001) both regarding prokaryotics [Figure 4(B)] and eukaryotics [Figure 4(F)]. Relative abundance of several prokaryotic genera was significantly affected (*p* < 0.01), including *Hydrogenophaga*, *Terrimonas*, *Pseudomonas*, *Limnohabitans*, *Algoriphagus*, *Methylophilus*, *Arcobacter*, *Acidothermus*, *Azovibrio*, *Desulfobulbus* and *Polynucleobacter* [Figure 4(C,D)] while there were no significant differences regarding eukaryotic genera [Figure 4(G,H)].

### River water can induce bacteriophages in a site‐specific manner

**FIGURE 5:**
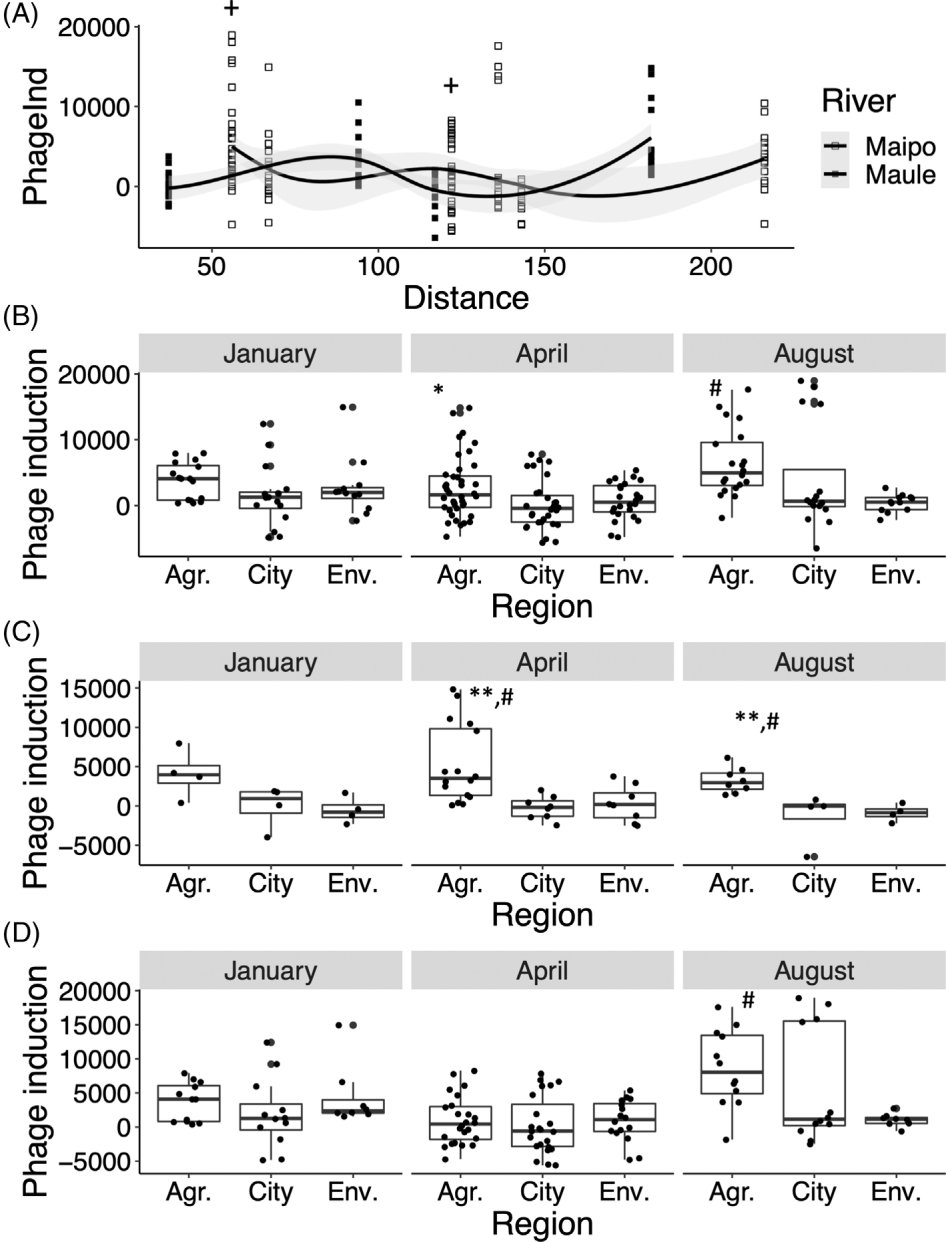
Seasonal and regional variation in phage induction in the rivers Maule and Maipo. Water collected from different river sites was added to an *E*. *coli* reporter system for bacteriophage induction (e.g. *recA* activation, SOS response) and induction measured as GFP levels, both for river Maule and Maipo (A). Induction was analysed based on regional variation according to agricultural areas (Agr.), city (City) or environment (Env.) for both rivers combined (B), river Maule (C) and river Maipo (D). Data were analysed by ANOVA followed by Tukey's test *post hoc*. *p < 0.05 and **p < 0.01, versus City. #*p* < 0.05 versus environmental locations. + denotes two sample sets from a similar distance (e.g. pre and post WWTP). The solid line shows the average phage induction and the grey shaded area the 95% confidence interval

Bacteriophages are known vectors of antibiotic resistance transmission, but their response to environmental stressors has not been thoroughly studied. Phage induction was found to vary statistically significant along the two rivers (Figure 5). This variation was explained by the region of sampling (agricultural, city, or environment; ANOVA *p* = 0.0021), distance along the river (*p* = 0.00074), sampling time (*p* = 0.00092) and the river itself (*p* = 0.048). Notably, the seasonal variation differed between rivers (interaction river*season, *p* = 0.00012).

Next, we assessed the regional variation in isolation. In Maule, agricultural areas showed higher phage induction than city (*p* = 0.00015) and environmental locations (*p* = 0.000039), independent of month. No such differences were observed in Maipo. Collectively, for both rivers, phage induction was higher in agricultural regions compared to cities (*p* = 0.018) and environmental locations (*p* = 0.017) for April and August, respectively [Figure 5(B)]. On an individual river level [Figure 5(C,D)], phage induction was higher in agricultural areas compared to environmental locations in both rivers in August. In addition, for Maule, the degree of phage induction in agricultural areas exceeded that of city and environmental locations for April; no difference between locations was observed for Maipo for this month.

### Antibiotic resistance genes are prevalent in both bacterial and phage populations

**FIGURE 6:**
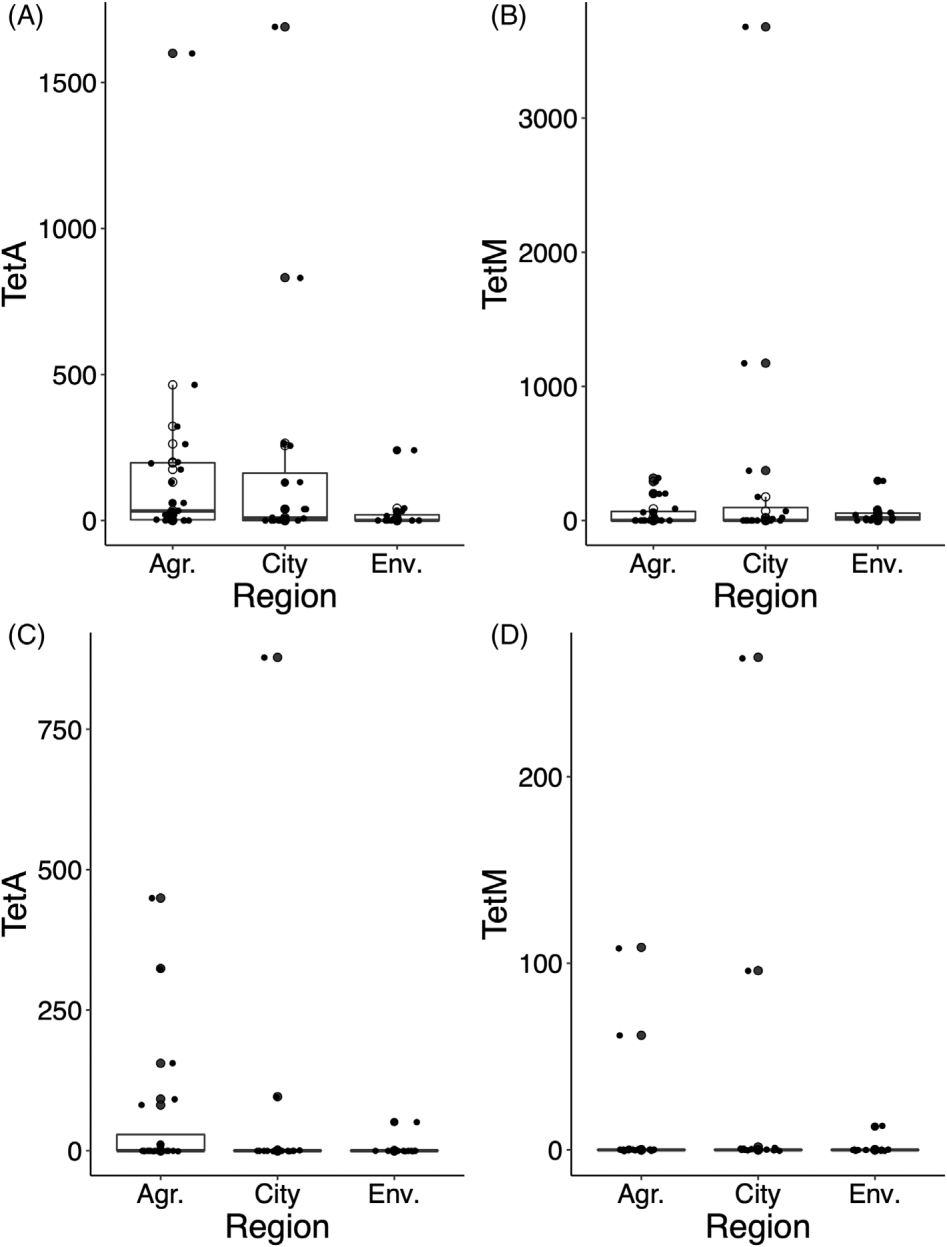
Antibiotic resistance genes are prevalent in bacterial and phage populations. River Maipo and Maule are illustrated with open and closed circles, respectively. (A) and (B), bacteria; (C) and (D) phage. *X*‐axis denotes number of gene copies in sample

A nanopore‐based metagenomic analysis of selected samples from a single season revealed high prevalence of *bla*TEM‐ and tetracycline genes, as well as a few macrolide and aminoglycoside‐related genes through mapping against the ResFinder database (data not shown). We, therefore, performed absolute quantification of a few chosen resistance genes (*tetA*, *tetM*, *ctxm1*, *ctxm9*) using ddPCR for the bacterial and the phage fractions. All samples were negative for the extended‐spectrum β‐lactamases (data not shown) but showed high prevalence of both *tetA* and *tetM* [Figure 6(A–D)].

**FIGURE 7:**
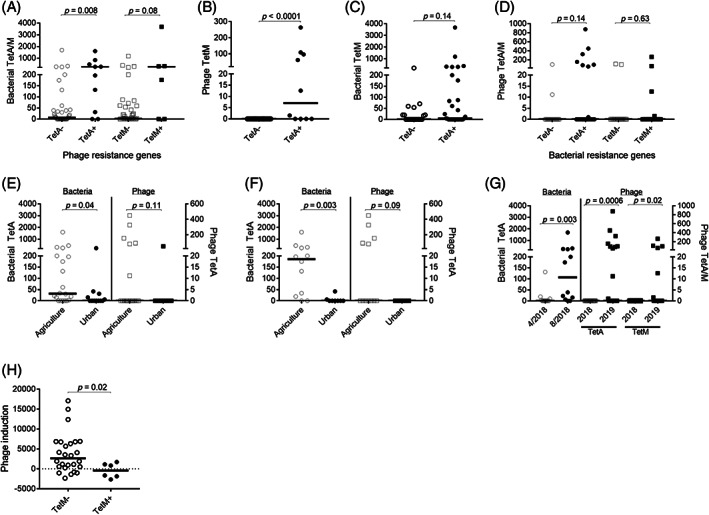
Presence of resistance genes in phages and bacteria. Presence (+) or absence (−) of phage resistance genes *tetA* and *tetM* in relation to (A) corresponding bacterial resistance genes, and (B) phage resistance gene *tetM*. Presence (+) or absence (−) of bacterial resistance gene *tetA* in relation to (C) bacterial resistance gene *tetM*. (D) Presence (+) or absence (−) of bacterial resistance genes *tetA* and *tetM* in relation to corresponding phage resistance genes. Presence of resistance gene TetA in bacteria and phages stratified on agricultural or urban setting in (E) all river systems, and in (F) the Maipo river. (G) Presence of resistance genes in bacteria and phages stratified on time‐point of collection. (H) Assessment of phage‐inducing ability stratified on presence of resistance gene *tetM* in phages. Statistical analyses were done using Mann–Whitney *U* test with bars representing the median value. *X*‐axis denotes number of gene copies in sample

Asking whether presence of resistance genes in phages would equate presence of resistance genes also in bacteria, we found that presence of *tetA* and *tetM* in phages corresponded to presence of *tetA* and *tetM* in bacteria [*p* = 0.008 and *p* = 0.08. respectively, Figure 7(A)], though not reaching statistical significance for *tetM*. Furthermore, *tetA* and *tetM* were often co‐expressed in phages, with *tetM* more so prevalent in phages having *tetA* expression [*p* < 0.0001, Figure 7(B)]. In contrast, for bacteria, there was no significant association between presence of *tetA* and *tetM* within bacteria [*p* = 0.13, Figure 7(C)], nor between bacteria and phages [*p* = 0.14 and *p* = 0.63, respectively, Figure 7(D)].

### Resistance genes vary due to biogeography

Considering environment as influencing prevalence of resistance genes, we next asked whether more resistance genes were to be found in an agriculture setting compared to a city. Our findings suggest that *tetA* is more prevalent in bacteria [*p* = 0.04, Figure 7(E)] but not in phages [*p* = 0.11, Figure 7(E)] in an agriculture setting, in particular for one of the rivers, Maipo [p = 0.004, Figure 7(F)].

Not only was expression of resistance genes affected by location but also by the time of collection. In particular, *tetA* expression in bacteria was higher in August 2018 compared to April 2018 [*p* = 0.003, Figure 7(G)]. Similarly, expression levels of *tetA*, and *tetM* in phages, were significantly higher in 2019 compared to 2018 [*p* = 0.0006 and *p* = 0.02, respectively, Figure 7(G)], consistent with an increase in prevalence of resistance genes over time.

Finally, we asked whether presence of resistance genes would influence the phage‐inducing capacity. Surprisingly, presence of *tetM* in phages was associated with reduced phage‐inducing capacity [*p* = 0.02, Figure 7(H)]. No statistical associations were seen between *tetA* expression and phage‐inducing capacity (not shown).

## DISCUSSION

Spread of antibiotic resistance has historically mainly been investigated in isolated systems within occupational settings (e.g. hospitals/patients, veterinary, environmental). With the advent of the One Health concept, these traditions have been integrated, allowing for a more translational view on transmission of resistance through different systems (Destoumieux‐Garzón et al., 2018). This concept has highlighted the importance of water as a vehicle for spread of resistance, with several papers demonstrating prevalence of resistance genes in contaminated rivers (Mittal et al., 2019; Posada‐Perlaza et al., 2019; Wu et al., 2020). However, resistance gene transfer is regulated by environmental changes (e.g. biogeographic variation) and what mechanisms regulate such transmission has not been thoroughly investigated.

Several studies have indicated the impact of subclinical levels of defined stressors (e.g. antibiotics, pollutants) for spread of antibiotic resistance genes through bacteriophages (Torres‐Barceló, 2018), with general air pollution suggested to be a factor that facilitates spread of resistance genes (Zhu et al., 2021). However, the impact of complex samples (e.g. river water, wastewater) (Posthuma et al., 2020), for such induction has not been thoroughly investigated. Here we show the phage‐inducive capacities of river waters and can for the first time demonstrate that this capacity is directly connected to the surrounding environment with farming land being associated with a higher inducive capacity.

Prevalence of resistance genes in agriculture has been demonstrated and reviewed; however, most of these papers disregard the putative impact of bacteriophages in such systems (Manyi‐Loh et al., 2018; Thanner et al., 2016). Manyi‐Loh et al. (2018) connect resistance spread within agriculture to the One Health concept and argue that regulations need to be strengthened to reduce antibiotics within agriculture to lower selective pressure on bacteria but also mainly disregard any possible impact of bacteriophages in such setting. Given the longevity of bacteriophages, able to persist for decades in different environments (Clokie et al., 2011), their importance in spreading resistance and serving as a reservoir for resistance genes cannot be underestimated, an opinion shared by Calero‐Cáceres et al. (2019) stressing that phages likely play a more ‘important role in the acquisition, maintenance and spread of ARGs than previously expected’ (Calero‐Cáceres et al., 2019). Several recent reports have identified resistance genes from bacteriophages isolated from water samples, including among other beta‐lactamases, multidrug efflux proteins and polymyxin resistance genes (Calero‐Cáceres et al., 2017, 2019; Colomer‐Lluch et al., 2011). Several of these genes could be transferred to bacteria and generate resistance, pointing towards the importance of resistance genes carried by bacteriophages for spread of resistance (Ross & Topp, 2015). While of high value, these studies are descriptive with no longitudinal samples collected for temporal variation. A Spanish study, one of few biogeographical studies, demonstrated a high prevalence of resistance genes in rivers and sediment that can be mobilized by phages (Calero‐Cáceres et al., 2017). The results generated in our study are in concordance with the main conclusions from Calero‐Cáceres et al., but further demonstrates the impact of stressors in water facilitating mobilization of phages as an important mechanism for antibiotic resistance spread.

We could detect a significant contribution of distance from water source to phage‐inducive capacity. It has been demonstrated that usage of manure or biosolids for fertilization of soil may increase abundance of bacteriophages and antibiotic resistance genes, as well as transduction events (e.g. spread of resistance genes) (Ross & Topp, 2015). Likewise, excessive usage of antibiotics in fish farms has been demonstrated to impact ARG abundance more than 100 m away (Bueno et al., 2019). It is thus possible that usage of either antibiotic‐like substances (e.g. pesticides, stressors) or ARG‐containing manure can impact local environments, allowing these to mobilize phages carrying resistance genes. Such potential impact would, however, mainly be local, as demonstrated by the articles above.

It should be noted that the method of choice for measuring phage induction is based on a bacterial reporter system, with a *recA* promoter fused to GFP. Thus, when exposed to stress, GFP will be expressed level of fluorescence correlated to induction of phage. However, water samples with limited nutritional value, and/or high concentrations of toxic substances (e.g. high stress) will result in killing of the reporter bacteria, and thus negative values for induction. Therefore, phage induction values deviating from zero (e.g. both highly positive and highly negative values) may result in phage induction in a natural biological setting. It is therefore not surprising that presence of *tetM* within phage capsids is significantly associated with a reduction in phage induction.

Resistance profiles and inducing capabilities vary with rivers, river sites, as well as with seasonal changes. These factors are, however, dependent on each other, for example seasonal changes affect levels of water in the rivers (rain, dryness), stress (UV from sun, concentration of stressors based on river level), as well as dictate the surrounding activities (harvest, farming) which in turn affect the concentration and composition of stressors in the rivers. It may therefore be difficult to specifically pinpoint what factor is the driving force in spread of resistance. However, it is clear that levels of stressors are affected by multifactorial conditions. Calero‐Cáceres et al. also studied seasonal changes of ARG in rivers and sediment but found no impact from rainfall, temperature in water, UV, pH, salinity or total organic carbon. Unable to identify the underlying molecular mechanism, they still found significant variation in ARG content from season to season, in particular in less abundant ARGs (Calero‐Cáceres et al., 2017).

Tetracycline resistance (*tetA*, *tetM*) serves as a biomarker for antibiotic resistance in this study. Resistance towards this drug has been identified in the environment, aquaculture, agriculture, veterinary and human health (Roberts, 1996) and is thus a relevant resistance gene from a One Health perspective. Besides resistance towards tetracycline, β‐lactamases were the most commonly identified resistance genes, which is also reflected in their abundant usage in veterinary applications, livestock production as well as in human clinics (Graham et al., 2016)—making them critical in resistance spread between environmental bacteria and human/animal pathogens.

Another factor affecting prevalence of specific bacteriophages is general microbiota composition. Our study describes biogeographic variation as a significant factor for microbial diversity and species composition. Others have indicated geomorphology as an important factor for microbial diversity in rivers (URycki et al., 2020), as well as distance from source being an important variable for alpha and beta diversity (Payne et al., 2017), a factor that was not significantly affecting diversity in our investigated system. Further studies focusing on soil microbial diversity have indicated that bacterial diversity is not associated with common variables (temperature, latitude, geographic distance) that we often associate with animal diversity. Rather, diversity is influenced by very local variables (pH, minerals, nutrients, toxins) in a ‘micro’ scale (Fierer & Jackson, 2006). A variable microbiota directly affects bacteriophages preying upon them and thus impacting their ability to spread resistance. As such, bacterial ecosystems are important cornerstones in spread of resistance through bacteriophages and their changes over time and over geographical sites are key to understand the spread of resistance. In this study, we opted for a 16S rRNA amplicon deep sequencing approach of all the samples rather than a metagenomics analysis as our research question was elucidated at a sufficiently detailed level using what 16S rRNA sequencing could offer. Furthermore, we complemented the data set with absolute quantification of selected antibiotic resistance genes, rather than the relative values generated through metagenomics. Due to the high variation in microbial composition, resistance gene abundance and phage activation in the samples between different river sites and seasons it is imperative to follow such changes closely in order to limit a negative impact thereof. A major concern is the high usage of antibiotics within the agricultural industry, leading to a selection of resistant bacteria as well as a mobilization of resistance genes. Though being environmental samples, these findings have direct implication to human health, in terms of the bacteria being able to transmit resistance genes to important human pathogens, thus causing infections that are difficult to treat. A better understanding of what is regulating both selection and spread of resistance in water samples is therefore of high relevance to explore. Similarly to the COVID‐19 measurement in WWTPs, a system to follow resistance spread should be encouraged.

This study demonstrates a significant biogeographic variation in microbial compositions in rivers, in a time‐ and region‐resolved setting. Importantly, agricultural regions showed a high propensity to stimulate bacteriophage induction and resistance transduction, while also demonstrating a high proportion of resistant bacteria. Both resistance profile of the microbiome and the microbial composition itself varies depending on time, region (e.g. agriculture) and biogeographic site in the river, indicating the dynamic regulation of microbial compositions and resistance profiles within such environments. The collected data serve as a valuable baseline for further comparative studies to relate to and contribute with an important piece of the puzzle that constitutes antibiotic resistance spread in a One Health perspective. Based on the findings from this study, we postulate that not only do we need to consider removing resistant bacteria from environmental sources (e.g. water) but we may also need to focus on bacteriophages carrying resistance genes. Further research will aim to investigate the nature of the stressors to gain a deeper mechanistic understanding of phage induction.

## CONFLICT OF INTEREST

The authors declare no competing financial interests.

## Supporting information


**APPENDIX S1** Supporting Information

## Data Availability

The data that support the findings of this study are available from the corresponding author upon reasonable request.
