# Supplementary material for: Biogeographical variation in antimicrobial resistance in rivers is influenced by agriculture and is spread through bacteriophages
Source: Environ Microbiol. 2022 Jul 7;24(10):4869–84. doi: 10.1111/1462-2920.16122 (PMC9796506; doi:10.1111/1462-2920.16122)
Supplement: Supplementary file 1 — APPENDIX S1 Supporting Information [file EMI-24-4869-s001.pdf]

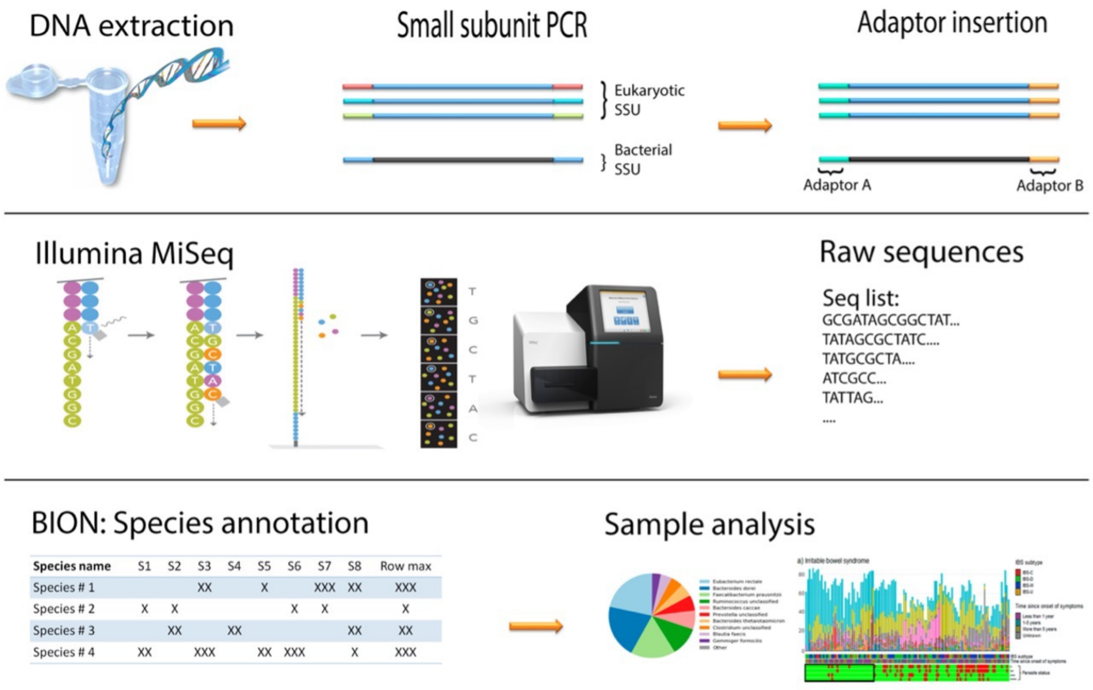

Figure S1. Microbiome workflow

DNA was extracted from all microbial samples, and a small subunit PCR performed with added adaptors for Illumina MiSeq sequencing generating raw sequences. The data was curated and species annotated, after which the samples were analyzed.

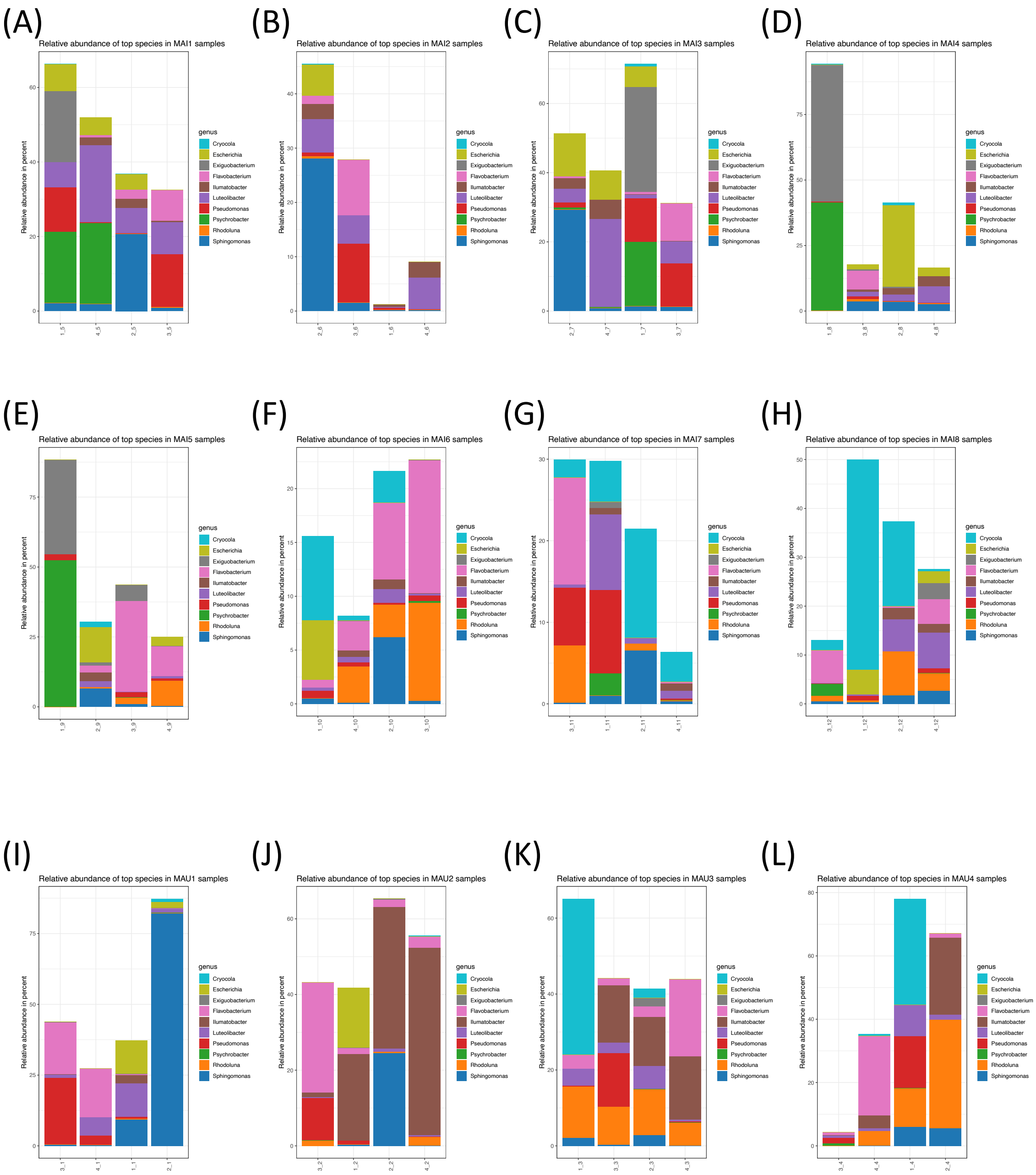

Supplementary Figure 2. Prokaryotic diversity at different river sites during different time points. Different river sites from both rivers (Maipo, n=8; Maule, n=4) were characterized for prokaryotic diversity, with the top 10 most common prokaryotic genera added in the graph. A) MAI1, B) MAI2, C) MAI3, D) MAI4, E) MAI5, F) MAI6, G) MAI7, H) MAI8, I) MAU1, J) MAU2, K) MAU3, L) MAU4. The first number in the sample ID denotes the time point of sampling, while the second number denotes the site.

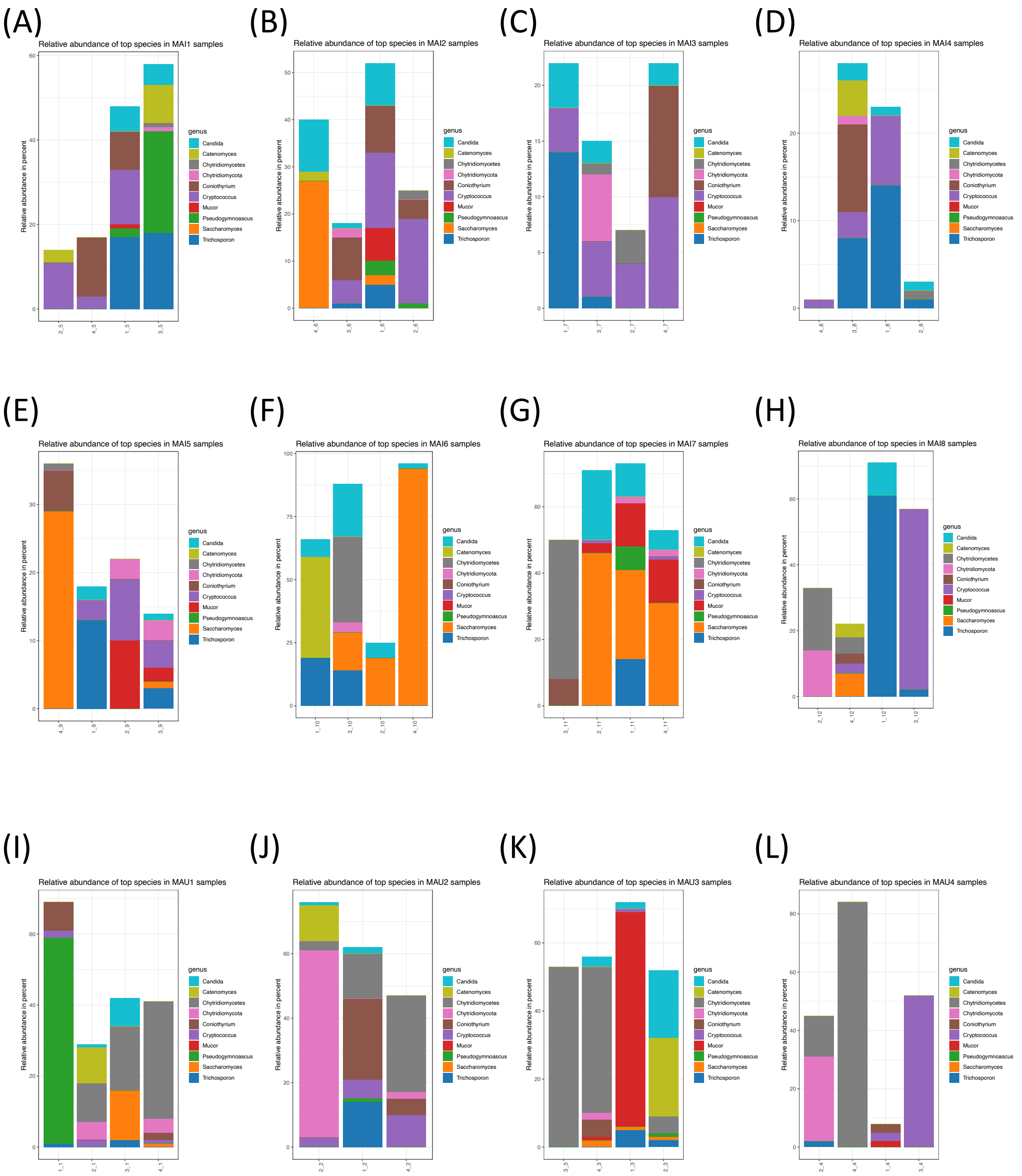

Supplementary Figure 3. Eukaryotic diversity at different river sites during different time points. Different river sites from both rivers (Maipo, n=8; Maule, n=4) were characterized for eukaryotic diversity, with the top 10 most common eukaryotic genera added in the graph. A) MAI1, B) MAI2, C) MAI3, D) MAI4, E) MAI5, F) MAI6, G) MAI7, H) MAI8, I) MAU1, J) MAU2, K) MAU3, L) MAU4. The first number in the sample ID denotes the time point of sampling, while the second number denotes the site.
